# The MAL Protein, an Integral Component of Specialized Membranes, in Normal Cells and Cancer

**DOI:** 10.3390/cells10051065

**Published:** 2021-04-30

**Authors:** Armando Rubio-Ramos, Leticia Labat-de-Hoz, Isabel Correas, Miguel A. Alonso

**Affiliations:** 1 Centro de Biología Molecular “Severo Ochoa”, Consejo Superior de Investigaciones Científicas and Universidad Autónoma de Madrid, 28049 Madrid, Spain; arubio@cbm.csic.es (A.R.-R.); llabat@cbm.csic.es (L.L.-d.-H.); isabel.correas@uam.es (I.C.); 2 Department of Molecular Biology, Universidad Autónoma de Madrid, 28049 Madrid, Spain

**Keywords:** condensed membranes, membrane trafficking, toxins, epithelial cells, T cells, myelin-forming cells, gene hypermethylation, cancer, MARVEL domain, biomarker

## Abstract

The *MAL* gene encodes a 17-kDa protein containing four putative transmembrane segments whose expression is restricted to human T cells, polarized epithelial cells and myelin-forming cells. The MAL protein has two unusual biochemical features. First, it has lipid-like properties that qualify it as a member of the group of proteolipid proteins. Second, it partitions selectively into detergent-insoluble membranes, which are known to be enriched in condensed cell membranes, consistent with MAL being distributed in highly ordered membranes in the cell. Since its original description more than thirty years ago, a large body of evidence has accumulated supporting a role of MAL in specialized membranes in all the cell types in which it is expressed. Here, we review the structure, expression and biochemical characteristics of MAL, and discuss the association of MAL with raft membranes and the function of MAL in polarized epithelial cells, T lymphocytes, and myelin-forming cells. The evidence that MAL is a putative receptor of the epsilon toxin of *Clostridium perfringens*, the expression of MAL in lymphomas, the hypermethylation of the *MAL* gene and subsequent loss of MAL expression in carcinomas are also presented. We propose a model of MAL as the organizer of specialized condensed membranes to make them functional, discuss the role of MAL as a tumor suppressor in carcinomas, consider its potential use as a cancer biomarker, and summarize the directions for future research.

## 1. Introduction

**Figure 1:**
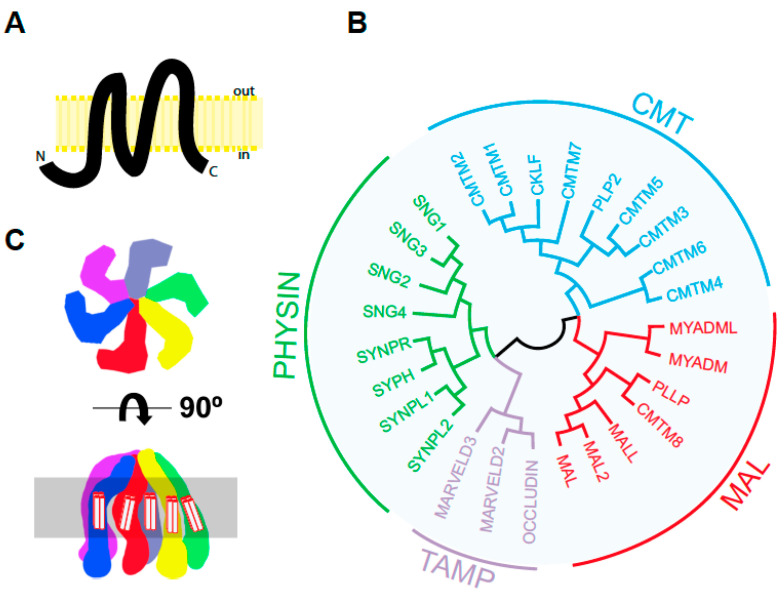
The human MARVEL superfamily of proteins. (**A**) Predicted structure of human MAL according to Uniprot (https://www.uniprot.org/uniprot/P21145, accessed on 5 April 2021). Protein segments: 1–24 cytoplasmic, 25–46 transmembrane, 47–53 first extracellular loop, 54–75 transmembrane, 76–92 cytoplasmic loop, 93–114 transmembrane, 115–124 second extracellular loop, 126–147 transmembrane, 148–153 cytoplasmic. The human MAL sequence as well as that of other species in shown in Figure S1. The length of the different segments in the schematics is drawn to scale. (**B**) Tree of human MARVEL domain-containing proteins. The sequence of the indicated proteins except their cytoplasmic amino- and carboxyl-terminal tails was aligned using the Muscle algorithm of the Jalview software [6], and the resulting alignment was used in MegaX [7]. The protein accession numbers of the corresponding sequences were: CMTM1 (NP_443725.3), CMTM2 (NP_653274.1), CMTM3 (NP_653201.1), CMTM4 (NP_848933.1), CMTM5 (NP_612469.1), CMTM6 (NP_060271.1), CMTM7 (NP_612419.1), CMTM8 (NP_849199.2), CKLF (NP_058647.1, PLP2/A4 (NP_002659.1), MYADM (NP_612382.1), MYADML2 (NP_001138585.2), MAL (NP_002362.1), MAL2 (NP_443118.1), MALL/BENE (NP_005425.1), PLLP/plasmolipin (NP_057077.1), occludin (NP_002529.1), MARVELD2/tricellulin (NP_001033692.2), MARVELD3 (NP_443090.4), SYPH/synaptophysin (NP_003170.1), SYPL1/pantophysin (NP_006745.1), SYPL2/mitsugumin (NP_001035799.1), SYNPR/synaptoporin (NP_001123475.1), SNG1 (NP_004702.2), SNG2 (NP_004701.1), SNG3 (NP_004200.2) and SNG4 (NP_036583.2). (**C**) Two views of the three-dimensional structure proposed for the hexameric synaptophysin complex. The highlighted area represents the lipid bilayer. The transmembrane segments of the MARVEL domain are indicated.

The human *MAL* cDNA was originally identified during a search for genes that are selectively expressed during T cell differentiation [1]. In addition to T cells, *MAL* gene expression was detected in specific polarized epithelia and in myelin-forming cells [2,3,4,5]. The *MAL* gene encodes a highly hydrophobic integral membrane protein of 153 amino acids that is conserved across vertebrates; approximately 53% and 69% of the residues in human MAL are identical or similar, respectively, to those in zebrafish MAL. Structural predictions suggest that MAL has a four transmembrane-helix architecture with its N- and C-terminal ends oriented towards the cytoplasm (Figure 1A) [1].

MAL shares significant sequence similarity in its four transmembrane domains with other integral membrane proteins. This allows the definition of a domain, called the MARVEL (MAL and related proteins for vesicle formation and membrane link) domain, that is present in 24 human proteins. These constitute the MARVEL superfamily and are conserved across species [8]. The MARVEL superfamily includes the CMTM (chemokine-like factor MARVEL transmembrane domain containing) [9], MAL [10,11], physin [12,13], and TAMP (tight junction-associated MARVEL) [14] families (Figure 1B). The MAL family consists of seven members. MAL2, MALL/BENE, PLLP and CMTM8 display a tetraspanning topology similar to that of MAL. The other two members, myeloid differentiation-associated marker (MYADM) and MYADM-like2 (MYADML2), contain an additional MARVEL domain and form an independent branch within the family. Synaptophysin, which is the most abundant protein in synaptic vesicles [15], is the only MARVEL superfamily member whose 3D structure has been determined so far [16]. Synaptophysin adopts a hexameric basket-like complex with six spokes each, corresponding to a synaptophysin subunit, with an extended, open conformation on the vesicle lumen and a closed end containing the N- and C-termini on the cytosolic side of the vesicle membrane (Figure 1C). This structure is consistent with the proposed role of synaptophysin as a component of the fusion pore during synaptic vesicle exocytosis [17,18]. It is currently unknown whether MAL or other members of the MARVEL superfamily have a similar hexameric structure.

In this review, we address the biochemical properties and expression pattern of the MAL protein, its function in specialized routes of membrane trafficking in T cells, epithelial cells and myelin-forming cells, and its role in the organization of specialized membranes. The role of MAL as a receptor for the *Clostridial* epsilon toxin, the epigenetic regulation of *MAL* gene expression in cancer, and the use of MAL as a cancer biomarker are also discussed. 

## 2. Tissue Specific Expression, Intracellular Distribution, and Biochemical Properties of MAL

### 2.1. MAL Expression Is Restricted to Specific Tissues and Cell Types 

The generation of a highly specific anti-MAL antibody mouse mAb 6D9 allowed MAL expression and distribution to be immunohistochemically analyzed in frozen or paraffin-embedded human tissue (Table 1) [19,20]. The analysis revealed that MAL is strongly expressed in the epithelial cells of distal, but not proximal, tubules and collecting ducts of the kidney, and in many types of polarized epithelial cells, such as those lining the follicle lumen in the thyroid or the prostate acini. MAL is also expressed in: the stratified squamous epithelium of the esophagus; crypt cells and brush border enterocytes in the gut; type 2, but not type 1, pneumocytes in the lung; and in the transitional epithelium of the urothelium, most prominently in the superficial layer. The thymus cortex, paracortical T lymphocytes of lymph nodes, and peripheral blood T lymphocytes, as well as myelin-forming cells in peripheral nerve endings are also positive for MAL expression. Sporadic cells in the thymus medulla and in the lymph node follicles also express MAL [19,20]. In contrast to the lack of MAL expression in endothelial cells, high endothelial venules endothelium is positive, with a characteristic apical distribution. This differential expression was confirmed by DNA microarray analysis [21]. MAL expression was undetectable in fibroblasts, muscle cells and endothelial cells. The expression of MAL in myelin in the peripheral and central nervous system was confirmed in rats and mice [22,23]. In addition, although it has not been as extensively studied in mice as it has in humans, the tissue distribution of MAL appears concordant in the two species except that, in mice, peripheral blood lymphocytes and their thymic precursors do not express MAL [23,24].

In conclusion, MAL is characterized by an expression pattern restricted to specific cell types and tissues.

### 2.2. MAL Is Mainly Found at the Plasma Membrane and Cytoplasmic Tubulovesicular Structures

A quantitative electron microscopy analysis of MDCK cells stably expressing c-myc-tagged MAL [25] indicated the presence of MAL at the plasma membrane (12% of the total label), including clathrin-coated pits and buds, and cytoplasmic structures (88% of the total label). Most of the cytoplasmic labeling occurred in uncoated tubular/vesicular structures (82% of the total), with smaller fractions in clathrin-coated structures (4% of the total), multivesicular endosomes (MVE; 1.4% of the total), and Golgi-associated structures (0.6% of the total). This subcellular distribution suggests that MAL might move between distinct compartments. This possibility was confirmed by tracking the movement of the subpool present in the plasma membrane, which revealed continuous shuttling of MAL between the plasma membrane and the Golgi [26]. 

### 2.3. MAL Has Unusual Biochemical Properties

#### 2.3.1. MAL Is a Proteolipid Protein

The term proteolipid was originally coined to designate a complex protein fraction present in brain myelin extracts that partitioned with myelin lipids in organic solvents, such as chloroform:methanol mixtures, which are commonly used to extract cell lipids [27]. Proteolipids later came to be operationally defined as membrane proteins from any source that display unusual lipid-like properties that make them partition into lipophilic solvents [28]. The proteolipid group includes proteins with very different structures and functions, including the proteolipid protein (PLP), which is a major component of myelin membranes, and the 16-kDa subunit of the Vo sector of the eukaryotic H^+^-pump V-ATPase [28]. The MAL protein, which displays a hydrophobicity profile quite similar to that of the 16-kDa Vo proteolipid, partitions selectively into the organic phase when cells are extracted with lipophilic solvents [29]. This unusual biochemical feature qualifies MAL for inclusion in the proteolipid group.

#### 2.3.2. MAL Selectively Partitions into Condensed Membranes

Studies in model membranes have shown that collective interactions between sterols and saturated lipids induce the formation of condensed yet fluid “liquid-ordered” (Lo) phases coexisting with “liquid disordered” (Ld) phases [30,31,32]. Membrane nanodomains—often referred to as “liquid ordered” (Lo)-like membranes, condensed membranes or rafts—are specialized cellular membrane subdomains whose physicochemical properties include dense lipid lateral packing, enrichment in sterols and saturated acyl chains, and a transient lifetime [33,34]. 

The tight packing of lipids in condensed membranes confers resistance to solubilization by nonionic detergents, such as Triton X-100, at low temperature [35,36]. Although the association of proteins with detergent-resistant membranes (DRMs) does not necessarily mean that the partitioning of those proteins in condensed membranes precedes experimental manipulation, since it can also arise as an artifact of the detergent extraction procedure [37], DRMs are enriched in proteins associated with condensed membrane subdomains in the cell. A short peptide motif (Leu-Ile-Arg-Trp, LIRW) situated close to the C-terminus and that is located at a cytoplasmic interface of the protein with the lipid bilayer is essential for partitioning MAL into DRMs [38]. Pulse-chase experiments indicated that whereas a MAL protein with an ILRW sequence fractionated into DRMs soon after biosynthesis, a similar protein ending with an ILKW sequence was found in the soluble fraction due to a trafficking defect that impedes the export of MAL from the ER [39]. These results argue against the possibility that the acquisition of insolubility by MAL is an artifact of the DRM isolation procedure, and suggests that intact MAL resides in compact membranes in the cell, even though MAL is compatible with both insoluble and soluble membranes. This dual compatibility is also supported by in vitro reconstitution experiments in artificial membranes [40]. In conclusion, the selective association of MAL with DRMs in all the cell types assayed appears to be a genuine feature related to its capacity to interact with a compact lipid environment. 

The high affinity of MAL for condensed membranes was confirmed in an in vitro system based on cell-derived giant plasma membrane vesicles, which separate into coexisting ordered and disordered lipid phases. In this system, MAL partitions into condensed domains in giant plasma membrane vesicles with an affinity greater than that of all the other transmembrane proteins analyzed and similar to that of prototypical raft markers, such as glycosylphosphatidylinositol (GPI)-anchored proteins and cholera toxin, which binds the GM1 ganglioside [41].

Membrane condensation can be measured in live cells using polarity sensitive dyes, such as laurdan (6-lauryl-2-dimethylamino-napthalene) [42,43]. These membrane probes do not preferentially partition into a particular type of membrane, but their fluorescence emission spectrum undergoes a red shift depending on the degree of condensation of the membrane environment in which they are inserted, making it possible to distinguish densely packed, ordered membranes from those with a loosely packed, disordered structure. Consistent with its selective partitioning into DRMs, the use of laurdan indicates that MAL selectively distributes in compact membranes in epithelial cells and T lymphocytes [44,45,46]. 

In summary, MAL displays unique features that impart selective preference for condensed membranes both in vitro and in live cells. Henceforth, we will use association with DRMs to refer to the partition of a molecule into the insoluble membrane fraction isolated by biochemical means, whereas distribution in condensed membranes will refer to the presence of a molecule in highly ordered membrane domains of the cell.

## 3. The Role of MAL in Epithelial Cells

Polarized epithelial cells transport newly synthesized proteins to the apical surface directly from the Golgi or through an indirect route, which consists of endocytosis of cargo at the basolateral surface and its posterior traffic in vesicular carriers across the cell, in a process known as transcytosis [47,48]. The observations that the apical membrane of epithelial cells is enriched in glycolipids relative to the basolateral surface and that the sorting of glycolipids and proteins takes place at the *trans*-Golgi network constitute the basis of the proposal of raft-mediated transport to the apical surface [49,50]. This hypothesis was supported by experimental evidence showing that artificial membranes consisting of glycolipids and cholesterol are insoluble in non-anionic detergents such as Triton X-100 [50,51], and by the observation that membrane proteins relying on the direct route, such as the influenza virus hemagglutinin (HA) and GPI-anchored proteins, become resistant to detergent solubilization during biosynthetic transport to the apical membrane [52,53]. In accordance with the original formulation of the raft-mediated model of apical transport [54], the association of glycosphingolipids with cholesterol generates raft platforms at the trans-Golgi network that are immiscible in the other phospholipid-enriched membranes. These platforms recruit specific proteins that are then transported in vesicular carriers made of raft lipids. 

### 3.1. MAL in Apical Transport in Cultured Cells 

In the original proposal of rafts as platforms for apical transport, the existence of a specialized protein machinery was postulated to make the rafts competent for transport. This machinery would consist of a minimal set of proteins to ensure the processes of vesicle formation, cargo recruiting, targeting and fusion to the apical surface [54]. One approach used to identify this machinery involved the sequencing of proteins present in detergent-resistant membrane fractions from total cellular membranes after protein separation by two-dimensional gel electrophoresis. Caveolin [55], VIP36 [56], annexin XIIIb [57], and MAL [4] were identified by this approach, and were proposed as candidates to be components of the apical transport machinery. Subsequently, it was shown that exogenous (HA and p75NTR) and endogenous (gp114) single-span transmembrane proteins, chimeric GPI-anchored proteins (YFP-GPI and gD1-DAF), and exogenous (thyroglobulin) and endogenous (gp80/clusterin) secretory proteins [58,59,60,61] require MAL expression to be transported to the apical surface of MDCK cells in an efficient manner. It is of note that one set of studies emphasizes that the kinetics of apical delivery of newly synthesized proteins, but not the targeting of apical proteins, was affected by MAL depletion, as they were found in the apical surface at steady state [59,60,61]. Another study, however, claimed that apical targeting was impaired, as the apical markers assayed concentrated at the Golgi and were nearly absent from the apical surface at steady state [58]. Whether these differences were due to the different systems used to knock down (KD) MAL expression—antisense oligonucleotides in the first case, and inducible expression of antisense RNA in the other—and/or the extent of MAL silencing has not been addressed. The slow kinetics of apical delivery observed in MDCK cells with depleted levels of MAL was recapitulated in epithelial Fisher rat thyroid cells [60], confirming the requirement of MAL for efficient apical transport by the direct route.

**Figure 2:**
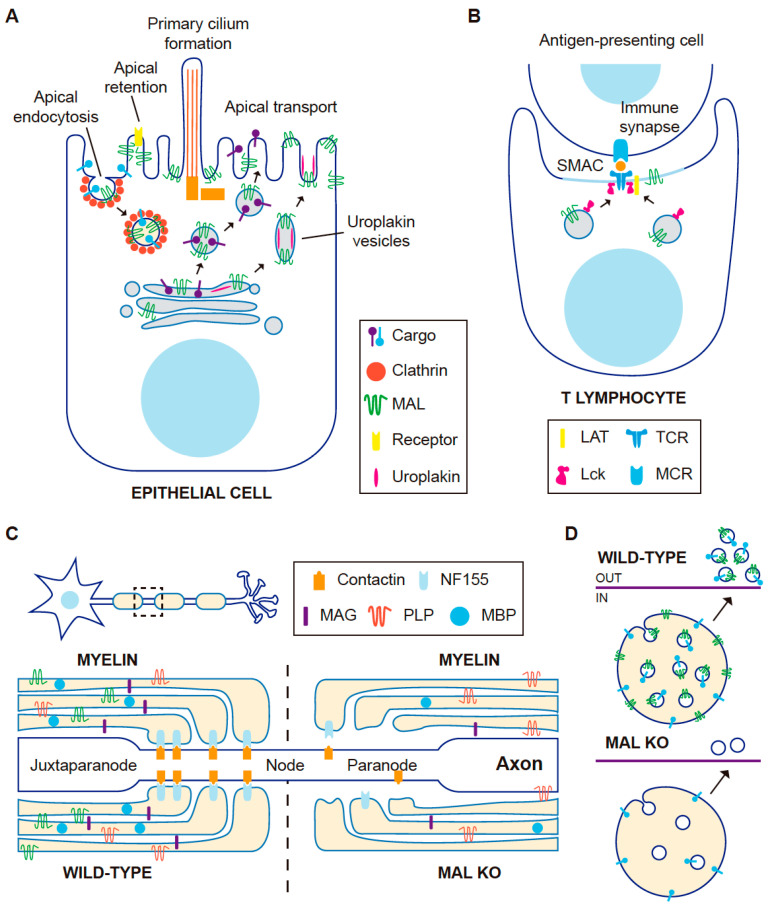
Function of MAL in different cell types. (**A**) In polarized epithelial cells, MAL has been involved in apical transport of specific cargo from the Golgi. In the case of urothelial cells, the vesicles transport preassembled urothelial plaque units. MAL is involved in a clathrin-dependent route of apical endocytosis, and in retention of specific proteins at the apical membrane. (**B**) In resting human T cells, MAL transports the tyrosine kinase Lck to the cell surface. In T cells conjugated with an antigen-presenting cell in the presence of antigen, MAL transports Lck to the immunological synapse. (**C**) In oligodendrocytes, MAL is important for proper architecture of nodes of Ranvier (boxed). Compared with wild-type mice, MAL KO mice show paranodal loops detached from the axon and reduced content of neurofascin (NF155), myelin-associated glycoprotein (MAG) and myelin basic protein (MBP) in myelin. (**D**) In T cells, and also probably in epithelial cells, MAL is important for generating intraluminal vesicles that will be secreted to the extracellular milieu as exosomes after fusion of the MVEs with the plasma membrane. In the absence of MAL expression, the release of exosome particles and markers is greatly reduced.

Unlike simple epithelial cells that directly transport GPI-anchored and single transmembrane domain proteins from the trans-Golgi network to the apical surface, in hepatocytes these proteins reached the apical membrane by basolateral-to-apical transcytosis [62]. Consistent with the absence of a direct route, MAL is not expressed in hepatocytes and hepatic cell lines such as HepG2 and WIF-B cells [63,64]. It is of note that GPI-anchored proteins and apical single transmembrane proteins do not partition into DRMs in control WIF-B cells but do so in WIF-B cells expressing MAL exogenously, and MAL reroutes them into the direct pathway [64]. These findings support the role of MAL as a component of the machinery of the direct route of apical transport mediated by raft membranes (Figure 2A).

### 3.2. MAL in Apical Transport: KO Mice

The apical surface of mammalian bladder epithelium contains one of the most effective permeability barriers known to exist in nature, whose function is to protect the epithelium from the urinary components. To accomplish this, the most superficial layer of urothelial cells is almost completely covered by large (0.10–1 μm) two-dimensional crystals of 16-nm uroplakin particles [65], known as urothelial plaques, comprising four major integral membrane proteins called uroplakins: tetraspanins Ia and Ib, and the single-span membrane proteins II and IIIa [66]. The intracellular trafficking of these plaques to the apical membrane is mediated by fusiform vesicles (500–800 nm), which consist of two plaques connected by a flexible, detergent-insoluble hinge region [67,68]. 

MAL expression is associated with in vitro urothelial cell differentiation [69]. Consistent with this finding, MAL is expressed in human [19] and mouse urothelium [70], as revealed by immunohistochemical analysis. As in other epithelial cells, MAL and uroplakins fractionate into DRMs of urothelial cells. In the superficial layer, MAL is present at the plasma membrane between adjacent plaques, and in the fusiform vesicles exclusively occupies the hinge region, which is the zone involved in fusion with the plasma membrane [70]. Consistent with the role of MAL in apical transport in MDCK cells, MAL knockdown (KD) decreases the rate of delivery, but not the targeting, of exogenous uroplakins to the apical membrane of MDCK cells. It is of note that urothelial cells from MAL knockout (KO) mice accumulate a large number of fusiform vesicles at their apical aspect, although the surface content of urothelial plaques is normal at steady state, indicating that the delivery but not the targeting of uroplakins is affected. In uroplakin-II KO mice, the formation of fusiform vesicles is blocked and, instead, vesicles of smaller size (~200 nm) appear that are positive for MAL but lack uroplakin plaques [70]. These findings suggest that, although MAL and uroplakins are able to form distinct types of vesicles in the absence of the other, they only form fusiform vesicles that are completely functional in plaque delivery when they are expressed together. The fact that MAL is present not only in the hinge areas of the fusiform vesicles but also in the hinge areas of the apical surface suggests that MAL might play a role in the fusion event leading to plaque delivery. This role would be consistent with the presence in MAL of a MARVEL domain, which, in synaptophysin, has been suggested to form part of a fusion pore complex that regulates synaptic vesicle exocytosis [17,18]. 

Similar to MAL, the v-SNARE protein VAMP8/endobrevin localizes at the hinge zone of fusiform vesicles [71]. Unlike MAL KO mice, which have a normal content of urothelial plaques at their apical surface, superficial urothelial cells from VAMP8 KO mice lack surface urothelial plaques but, as in MAL KO mice, form morphologically normal fusiform vesicles that do not fuse with the apical membrane [71]. Therefore, MAL appears to facilitate SNARE-mediated fusion of fusiform vesicles with the apical surface for efficient urothelial plaque delivery (Figure 2A). 

### 3.3. MAL in Apical Endocytosis

Since MAL continuously internalizes from the apical membrane [26], it was of interest know whether its internalization is used for the endocytosis of apical proteins. Consistent with this possibility, MAL and apical polymeric immunoglobulin receptor were visualized in the same endocytic vesicles by videomicroscopy, and were identified in the same clathrin-coated pits and vesicles by electron microscopy. Moreover, MAL silencing experiments indicated that apical, but not basolateral, endocytosis of the receptor requires MAL and clathrin. In contrast, the GPI-anchored folate receptor internalizes from the apical membrane in a MAL and clathrin-independent manner [72]. Therefore, in addition to controlling the traffic of proteins from the Golgi to the apical surface, MAL regulates a specialized pathway of endocytosis from the apical surface (Figure 2A).

### 3.4. MAL in Apical Retention of Membrane Proteins 

In the human kidney, MAL is expressed in distal tubules, Henle’s loop and collecting ducts, but not in proximal tubules and glomeruli [19]. The aquaporin-2 (AQP2) water channel is expressed exclusively in epithelial cells of the renal collecting duct [73]. AQP2 regulates water reabsorption by redistributing itself between intracellular vesicles and the apical cell surface in a process controlled by the antidiuretic hormone arginine vasopressin [74]. The renal-specific Na^+^-K^+^-2Cl^−^ cotransporter (NKCC2) is expressed in epithelial cells of the ascending limb of Henle’s loop. NKCC2 is responsible for Na^+^ and Cl^−^ reabsorption and the maintenance of normal blood pressure. Consistent with the absence of MAL in proximal tubules [19], LLC-PK1 cells, which derive from this type of structure, do not express MAL endogenously [75]. Ectopically expressed tagged MAL in LLC-PK1 associates with AQP2 and NKCC2 and causes their retention at the apical surface by decreasing their internalization. This observation was interpreted as indicating that MAL has a role in the apical retention of membrane proteins (Figure 2A) [75,76]. However, the overexpression of MAL, the presence of a tag in MAL, and the use of a cell line that does not express endogenous MAL limit the validity of this conclusion. Further work in renal epithelial cells positive for endogenous MAL expression, the analysis of the effect of MAL silencing, and the use of exogenous MAL expression in rescue-of-function experiments are needed to provide more compelling evidence that MAL plays such a role.

### 3.5. MAL in Apical Lumen Formation 

The formation of an apical lumen is a key step during epithelial tissue morphogenesis [77,78]. Lumen-containing organs are spherical, as in the case of the thyroid follicles, or tubular, as in the case of the branched tubules of mammary glands or lungs or the unbranched ones of the sweat glands. Defects in lumen formation are associated with a variety of disorders such as polycystic kidney disease and vascular stenosis, which are characterized by abnormal dilation of renal tubules or a reduction in the lumen size of blood vessels, respectively [79].

Mice overexpressing MAL develop multiple large renal cysts, with a close correlation between MAL expression levels and cyst development, the affected tubular segments expressing the highest levels of MAL. Detailed analyses revealed that the cysts probably developed from the distal convoluted and connecting tubuli, whereas proximal convoluted tubuli appeared to be undamaged [80]. MDCK cells have been widely used as a cell model system to study the process of single lumen formation [47,81]. When these cells are cultured in a three-dimensional matrix such as Matrigel^®^, they form spheres that progressively mature to form hollow structures, referred to as cysts. These consist of a single layer of epithelial cells with their apical surfaces lining an inner single lumen, and their basolateral surface contacting the extracellular matrix. Defects during this process lead to the formation of multiple lumens of smaller size [79]. Oligonucleotides designed to knock down MAL expression [82], as well as tagged-MAL overexpression [83], produce cysts with multiple lumens in MDCK cells. Since MAL overexpression produces dilated apical surfaces in MDCK cells [58], it is plausible that that the effect of MAL overexpression on the formation of multiple lumens in MDCK cells or of renal cysts in mice arises from the production of a large excess of apical membrane. However, it remains unclear how MAL silencing produces the same effect. In conclusion, although the exact role of MAL in the process is still unknown, fine-tuning MAL levels appears to be important for normal cystogenesis in renal cells.

### 3.6. MAL and Primary Cilium Biogenesis

The primary cilium is a single appendage that protrudes from the cell surface of most mammalian cells. It is made up of a ciliary membrane that surrounds a microtubule-based scaffold, known as the axoneme, that is derived from the older centriole in the centrosome [84]. Primary cilia recognize a wide range of environmental signals and transmit them to the cell body [85]. Defects in primary cilium functioning are associated with a long list of developmental and degenerative disorders [86,87]. 

In MDCK cells, MAL accumulates predominantly at the base of the cilium in moderately confluent cultures and also at the primary cilium at high cell density [46]. MAL KD results in a drastic drop in the percentage of ciliated cells [46,82]. The components of the machinery for ciliary growth are recruited normally to the centrosome zone under those conditions but are unable to elongate the primary cilium correctly and, consequently, the remaining cilia are stunted [46]. This effect of MAL silencing seems to be due to deficient condensation of the centrosome-associated membranes needed to build the ciliary membrane [88,89]. The overexpression of tagged MAL in MDCK cells and in transgenic mice also gives rise to fewer, shortened cilia. This may have been caused by excessive condensation of the ciliary membrane precursor associated with the centrosome, although this effect was not investigated at the time. In summary, MAL levels modulate proper primary cilium formation and elongation in renal cells (Figure 2A).

## 4. MAL in T Lymphocytes 

### 4.1. MAL Expression in T Lymphocytes

Flow cytometry analysis with anti-MAL mAb 6D9 indicates that MAL is strongly expressed in thymocytes, in a large percentage (65–90%) of peripheral CD4 T cells, and in a lower proportion (31%) of CD8 peripheral T cells. Consistent with the absence of MAL expression in B cell lines [1], CD19^+^ B cells were negative. MAL is not significantly expressed in peripheral blood B cells or in B cell subsets of tonsil and spleen [20].

Engagement of the T cell receptor (TCR) triggers a program of activation of gene expression. Costimulatory molecules provide additional signals that amplify those of the TCR and determine T cell fate. As a result, T cells undergo anergy, apoptosis, clonal expansion, or differentiate into helper T (Th) or regulatory T cells (Treg) cells [90]. CD28 and inducible costimulatory (ICOS)/CD278 are two costimulatory molecules of the same family that have distinct in vivo functions [91]. In a study to compare the programs of gene activation elicited by CD28 and ICOS costimulation, MAL was found among the small number of genes whose level of activation was higher in the case of ICOS costimulation, suggesting that MAL gene expression is regulated in different ways by CD28 and ICOS [92]. Depending on how IL-2 and interferon-α and IL-4, IL-5 and IL-13 are expressed, Th cells are subdivided into Th1 and Th2, respectively [93]. Atopic diseases, such as asthma, atopic dermatitis and rhinoconjunctivitis, are associated with Th2-driven inflammatory responses to common allergens. MAL was one of the genes found to be upregulated in the T cells of atopic disease patients compared with controls [94,95,96]. A subsequent study indicated that IL-4 expression is necessary to activate *MAL* gene transcription, prompting the authors to hypothesize that MAL might be involved in Th2 differentiation [96]. 

### 4.2. MAL on Vesicular Traffic of the Tyrosine Kinase Lck 

Endogenous and exogenous MAL selectively partitions into DRMs from human T cell lines and peripheral blood T cells [97,98]. MAL was found to be >10^4^-fold enriched in DRMs from the human HPB-ALL T cell line, similar to CD55, which is associated with the exoplasmic leaflet of the plasma membrane by a GPI-anchor. Its level is higher than that of the non-receptor Lck tyrosine kinase (~400-fold), which is associated with the inner leaflet through myristic and palmitic acid chains that are covalently attached to its N-terminal end. MAL cofractionates with Lck in the DRM fraction obtained from endosomal membranes, and both proteins colocalize in endosomal structures in Jurkat T cells and human peripheral blood T lymphocytes [98]. MAL and Lck travel in the same transport vesicles following microtubule tracks leading towards the plasma membrane [45]. The movement of these vesicles requires the MAL-associated formin INF2 [99]. In the absence of MAL, Lck accumulates in internal membranes and does not reach the plasma membrane efficiently. As a consequence, TCR-mediated cell signaling, which is known to require plasma membrane-associated Lck, is impaired, which is an indication of the importance of the role of MAL in human T cells [24]. In a recent study based on the use of a new mAb (MT3) that was generated to CD3-coprecipitated complexes, and that stains the T-cell surface only when MAL is expressed, it was suggested that MAL is involved in T cell differentiation but not in Lck transport [100]. However, since biochemical evidence showing that mAb MT3 recognizes MAL is lacking, it cannot be ruled out that mAb MT3 recognizes a protein whose transport to the cell surface is dependent on MAL, expression or an epitope associated with the TCR/CD3 complex that is unmasked only in the presence of MAL. It is of particular note that HIV-1 Nef specifically hijacks the MAL-mediated route of Lck transport by associating with MAL and the small GTPase Rab11b, leading to intracellular accumulation of Lck [101]. In conclusion, MAL has a crucial role as a component of the Lck transport machinery in T cells [102]. 

### 4.3. Subcompartmentalization of the Immunological Synapse

T lymphocytes polarize when they recognize an antigen presented by an antigen-presenting cell, forming a specialized domain, known as the immunological synapse (IS), at the zone of membrane contact between the two cells, and reorient the centrosome to face the IS. The IS consists of a supramolecular activation complex (SMAC) that is organized into two concentric membrane subdomains: a central complex (cSMAC) made up of the TCR, Lck and the transmembrane LAT adapter, and a peripheral ring (pSMAC), comprising cytoskeletal components and adhesion molecules [103]. In the absence of MAL, T cells form an IS but the neither the TCR nor Lck are targeted to the cSMAC and the centrosome does not reorient [24], suggesting that MAL has an important role in IS assembly (Figure 2B). 

Further evidence of the role of MAL in the correct assembly of the IS comes from the finding that the expression of a MAL molecule artificially targeted to the pSMAC missorts Lck and LAT to the pSMAC, and redistributes the docking machinery for transport vesicles and microtubules and transport vesicles from the cSMAC to the pSMAC [45]. Once MAL has delivered Lck to the IS, synaptic Lck, in turn, regulates the fusion of LAT- and TCR-containing transport vesicles to the IS to complete the assembly of the IS, thereby enabling TCR-mediated signaling [24,104]. Therefore, MAL is at the top of a hierarchically organized set of membrane trafficking pathways that delivers Lck to the IS and that enables the sorting and transport of other relevant components to the cSMAC.

## 5. MAL in Myelin-Forming Cells

### 5.1. The Role of MAL in Myelin Proteolipid Protein (PLP) Targeting to Myelin in Cultured Cells

Oligodendrocytes and Schwann cells produce vast amounts of myelin, a multilamellar membrane that wraps the axons of the central and peripheral nervous system (CNS and PNS), respectively. The myelin sheath is continuous with the plasma membrane and forms specialized subdomains with distinct lipid and protein composition that are generated and maintained by specialized processes of membrane trafficking [105,106]. MAL expression in oligodendrocytes was identified by differential screening of an oligodendrocyte cDNA library [3], and by the sequencing of proteins present in the DRM fraction of oligodendrocytes [2]. In situ hybridization techniques indicated that MAL is expressed not only in oligodendrocytes but also in Schwann cells [3]. Consistent with this finding, immunohistochemical [3,22,107], cell fractionation [108], and immunoblot analyses indicate the presence of MAL in myelin from both the central and peripheral nervous systems [3,23,108]. 

MAL is expressed in the CNS of rats during the late stages of myelination, whereas it is detected prior to the onset of myelination in the PNS. This observation led to the suggestion that MAL plays different roles in oligodendrocytes and Schwann cells. Thus, the late expression of MAL protein in the CNS is consistent with a role for it in the final stages of myelin sheath formation, such as the stabilization of the compacted myelin membranes, whereas the early expression of MAL protein in the PNS suggests that MAL is involved in Schwann cell differentiation and the onset of myelination [22]. In situ hybridization revealed that *MAL* is expressed in the human CNS and especially in the grey matter of the cerebral cortex, with less in the cerebellum and the amygdala and little or none in the subcortical white matter [109]. NTERA2 cells, which were isolated from the TERA2 teratocarcinoma-derived cell line, differentiate into neurons and other cell types when exposed to retinoic acid. Further evidence of the expression of MAL in neurons in the CNS comes from the finding that treating NTERA2 cells with retinoic acid induces MAL expression [109]. Comparative immunohistochemical analysis of human and rat brain needs to be performed to establish whether there is species-specific expression of MAL in CNS neurons.

Primary oligodendrocytes in monoculture express myelin components and form highly branched myelin-containing structures, called myelin sheets [110]. HA [111] and the major myelin-specific proteolipid PLP fractionate into DRMs in these cells and appear to use a pathway reminiscent of the sphingolipid-mediated route of apical transport to the cell body plasma membrane [111,112]. PLP, but not HA, is then transported by transcytosis to myelin sheets, in a process probably mediated by the MAL family member MAL2 [63,113], after having undergone a conformational change triggered by sulfatide that forces PLP to switch from being Triton X-100-insoluble to a Triton X100-soluble and CHAPS-insoluble form [114]. MAL overexpression does not affect the initial transport of PLP to the cell body plasma membrane but interferes with the conformational change required for the transcytotic transport of PLP to the myelin sheets. This facilitates lateral diffusion of PLP directly from the plasma membrane to the myelin membrane [112]. Therefore, MAL appears to switch the mode by which PLP reaches the myelin sheath from one of transcytosis, when MAL is not yet expressed, to one of lateral diffusion, during the late stages of myelinization when MAL is expressed. Since sulfatide triggers the conformational change of PLP, and MAL is associated with sulfatide [107], it is plausible that MAL mediates this switch by associating with sulfatide and impeding the conversion of PLP to the Triton X-100-soluble and CHAPS-insoluble form. The switch in the mechanism of PLP access into myelin might be important during development, since MAL expression takes place when myelin compaction has already started to make vesicular trafficking difficult [112]. Septins, which are guanine nucleotide-binding proteins that form high-order polymers [115], interact with MAL, and this interaction could be involved in structuring the myelin membrane [116]. 

Metachromatic leukodystrophy is a lysosomal lipid storage disease caused by arylsulfatase A deficiency, which leads to sulfatide accumulation and thereby progressive demyelination [117]. In arylsulfatase A-deficient mice, a mouse model of the disease, the accumulation of sulfatide occurs not only at lysosomes but also in myelin itself. It causes a dramatic reduction in MAL levels, but does not affect the overall composition of myelin [118]. This effect, together with the observation that sulfatide accumulation diverts MAL to the late endosomal/lysosomal compartment, reinforces the link between sulfatide and MAL and suggests that deficient MAL levels and/or MAL mistargeting may contribute to the pathogenic mechanism of metachromatic leukodystrophy. 

### 5.2. MAL in Myelin-Forming Cells in Mice

The role of the MAL protein in myelin-forming cells has also been studied in mice that overexpress or lack MAL expression [80,119,120]. Transgenic mice that express an excess of MAL exhibit delayed onset of myelination and progressive segregation of unmyelinated axons in the PNS, and hypomyelination and aberrant myelin formation in the CNS, which is evidence of MAL’s role in myelin formation [80]. The expression of the p75 neurotrophin receptor gene, which mediates the axon–glia interaction during ensheathment and myelin wrapping, is reduced in peripheral nerves of mice that overexpressed MAL [120]. Compared with wild-type mice, shorter intermodal length was observed in the peripheral nerves of MAL overexpressing mice, while it was longer in MAL KO mice [120]. This finding indicates that MAL expression affects the longitudinal extension of the myelin sheath. MAL overexpression alters the expression of a number of genes that influence the cytoskeletal organization and differentiation of Schwann cells [121], helping to explain the effect observed in PNS myelination. In the CNS, the organization of Ranvier nodes is defective in MAL KO mice, mainly in the paranodal zone [119], which is the zone where myelin anchors to the axon [122]. The incorporation of the paranodal protein neurofascin 55 and other myelin proteins is reduced (Figure 2C), explaining the anomalies observed in myelin organization [119]. In conclusion, in the CNS, MAL plays an important role in axon–glia interaction in the adult whereas MAL influences the expression of components that mediate this interaction during ensheathment and myelin wrapping in the PNS.

## 6. Role of MAL in Exosome Secretion 

Exosomes are extracellular vesicles, 50–100 nm in length, that arise from the discharge of preformed intraluminal vesicles within MVEs to the cell exterior by fusion with the plasma membrane [123]. Exosomes play an active role as vehicles for transporting a wide range of bioactive molecules (receptors, enzymes, cytokines, mRNA, miRNA, etc.) between different cells and tissues to modulate their activity. This important function represents a general mechanism of intercellular communication that controls development and a wide range of normal and pathological processes [124]. 

### 6.1. MAL in Prostasomes

Prostasomes are specialized exosomes secreted by the prostate gland involved in the immune protection of sperm cells and promotion of the fertilizing ability of sperm cells [125]. MAL localizes to multivesicular endosomes that contain other MAL family proteins, GPI-anchored proteins and caveolin-1, and is found in prostasomes secreted by prostate cancer PC-3 cells [126]. Since prostasomes are highly enriched in raft lipids [127], it is plausible that MAL participates in prostasome biogenesis. However, the direct involvement of MAL in this process has not yet been addressed. 

### 6.2. MAL in Exosome Biogenesis by T Lymphocytes

Videomicroscopic analysis of Jurkat T cells revealed the presence of MAL in large endosome structures that were also positive for the tetraspanin protein CD63, a prototypical marker of exosomes [128]. Some of these endosomes were found to move towards the plasma membrane and discharge their content into the extracellular space. Evidence of the involvement of MAL in exosome biogenesis came from the reduced number of exosomes secreted in MAL KD cells, as assessed by the direct visualization of exosome particles by electron microscopy, and the decreased content of exosome cargo, as demonstrated biochemically. The effect of MAL KD on exosome production is correlated with reduced sorting of CD63 into the intraluminal space of MVEs and with the accumulation of CD63 on the limiting membrane of aberrant MVEs that no longer fuse with the plasma membrane but, instead, merge with autophagic vacuoles and lysosomes [128]. Ceramide is important for the formation of intraluminal vesicle destined for exosomes in T cells, given that the silencing of neutral sphingomyelinase II or inhibition of its enzymatic activity impairs exosome release [129]. Since the effects of the inhibition of ceramide synthesis and of MAL depletion are similar, it was proposed that MAL organizes ceramide patches at the MVE-limiting membrane to promote cargo selection, and the inward invagination of the patches leading to the formation of intraluminal vesicles destined for exosome secretion (Figure 2D) [130]. 

Expression of the HIV-1 accessory protein Nef induces massive secretion of exosome markers in T cells [129]. Consistent with the involvement of MAL in exosome secretion in T cells, the release of the exosome markers was resilient to Nef expression in MAL KD T cells [128]. This finding supports the role of MAL as a critical component of the machinery for exosome biogenesis by human T cells.

## 7. MAL in Other Cell Systems and Processes

Interleukin (IL)-11, which is a member of the IL-6 family, exhibits pleiotropic functions, including hematopoiesis, bone development, tissue repair, and tumor development [131]. Human umbilical cord blood mononuclear cells are a rich source of stem and progenitor cells that could be of interest in repairing perinatal brain damage [132], and acute myocardial infarction treatment [133]. IL-11 induces MAL expression in human umbilical cord blood mononuclear cells [134], consistent with MAL playing a role in tissue repairing by these cells. 

Membrane-type 1 matrix metalloproteinase (MMP1) promotes cell migration and invasion and is involved in cancer cell invasion, metastasis and growth, angiogenesis, epithelial morphogenesis, skeletal development, inflammation, atherosclerosis, obesity, and rheumatoid arthritis [135]. MMP1 associates with MAL in co-immunoprecipitation experiments in COS cells that overexpress the two proteins. It is interesting to note that MAL expression reduces the invasion and migration capacity of MMP1-transfected cells, suggesting that MAL might regulate MMP1 activity [136]. Although it is of interest, the physiological role of this interaction is not known.

Maternal viral infections are recognized to have detrimental effects on the human fetus and serious consequences for the development and viability. A link has been established between maternal influenza infections and pre-term birth, low birth weight, congenital defects of the heart, cardiovascular disease as adults, and neurological and behavioral abnormalities [137]. In mice, influenza virus infection impairs the development of the fetal immune system and causes the downregulation of the expression of the *MAL* gene in developing fetal thymus [138]. However, since MAL is expressed neither in adult thymus nor peripheral T cells in mice [45], it is not clear that this effect is related to the observed defects in immune system development.

In HOG oligodendroglioma cells, herpes simplex virus type 1 particles colocalize partially with exogenous MAL [139]. Moreover, MAL depletion reduces the cell-to-cell spread of herpes simplex virus 1. This finding prompted the authors to suggest that MAL is necessary for the efficient spread of the virus [140]. However, as endogenous expression of MAL in HOG cells was not demonstrated and, consequently, the silencing of endogenous MAL could not be directly assessed, this hypothesis awaits confirmation. 

## 8. MAL and the Clostridial Epsilon Toxin

*Clostridium perfringens* types B and D produce epsilon-toxin (ETX), which is a potent pore-forming toxin that is responsible for devastating enterotoxemia in ruminants, which causes important economic losses. The toxin is produced in the gut of these animals and crosses the gut wall to enter the bloodstream, where it binds with great specificity to a restricted number of organs and structures, such as kidney, lung, blood–brain barrier endothelial cells and myelin. It causes perivascular edema in various tissues, accumulates in the kidney, and produces blood–brain barrier dysfunction and white matter injury [141]. ETX has been proposed as a possible environmental trigger for multiple sclerosis [142], a human inflammatory disease in which the blood–brain barrier is disrupted and myelin-forming cells of the CNS are injured [143,144], in a similar way to the alterations occurring during ETX-produced enterotoxemia in livestock.

ETX oligomerizes and forms channels in sensitive cell lines such as MDCK [145,146]. In these cells, ETX binds to condensed domains of the plasma membrane [147], is recovered in DRMs after binding to the cell surface [148], and binds to DRMs and to lipids extracted from them [149]. In overlays assays, ETX binds to sulfatide and phosphatidylserine [149]. Sulfatide is necessary for ETX cytotoxicity but not for binding to MDCK cells, suggesting a role for sulfatide in pore formation but not in binding to the target membrane [149]. It is of note that MAL was shown to bind sulfatide [107]. MAL and galactosylceramide sulfotransferase, which is the enzyme that converts galactosylceramide into sulfatide, are both regulated by thyroid hormone [150,151]. These findings are consistent with the existence of a link between MAL and sulfatide, and raise the possibility of a connection between MAL and ETX. 

Binding and functional experiments involving MAL KD in cells positive for endogenous MAL expression, and exogenous expression of MAL in cell lines that do not express endogenous MAL, together with co-immunoprecipitation of MAL and ETX, revealed that MAL appears to function as a receptor for ETX [152,153,154,155]. However, there is currently no evidence of a direct protein–protein interaction between MAL and ETX. MAL KO mice show the absence of ETX binding to tissues that bind the toxin and exhibit complete resistance to ETX at doses 1000-fold greater than those needed to cause ETX-associated symptoms in wild-type mice [152]. ETX sensitivity depends on oligodendrocyte expression of MAL, as MAL-deficient oligodendrocytes are insensitive to the toxin. In addition, ETX binding to white matter follows the spatial and temporal pattern of MAL expression [156]. ETX does not bind to wild-type zebrafish endothelial cells and, consequently, the toxin has no effect. However, a humanized zebrafish model expressing human MAL in endothelial cells binds to ETX and responds by producing blood–brain barrier leakage, blood vessel stenosis, perivascular edema and blood stasis [157]. In mice, MAL expression was found in the endothelium of some lung vessels, supporting the hypothesis that MAL is a key element in the ETX-induced perivascular edema in the lungs [158]. ETX binding is clearly observed in the microvasculature of the central nervous system but not in the vasculature of peripheral organs in the mouse, indicating that ETX specifically targets the endothelial cells of the CNS. ETX binding is dependent on MAL expression, since no binding to the CNS microvasculature of MAL KO mice was detected. It has been proposed that the effect of ETX on blood–brain barrier permeability in mice involves caveolae-dependent internalization induced by the binding of ETX to MAL at the vessel lumen membrane, and that this triggers the fusion of internalized caveolae with endosomes, the formation of MVBs, their transport across the endothelial cell and, finally, the possible release of exosomes into the opposite membrane, at the brain parenchyma [159]. 

**Figure 3:**
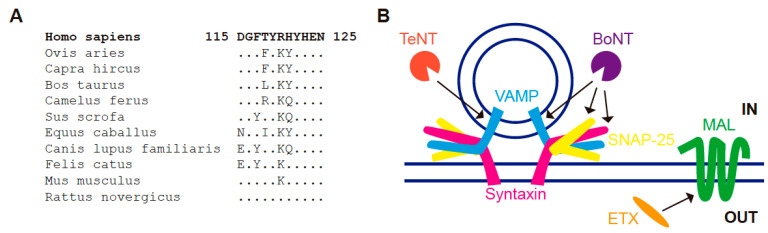
MAL as putative receptor of *Clostridial* ETX. (**A**) Sequence alignment of the predicted second extracellular loop of human MAL (*Homo sapiens*, accession number P21145) with that of different ruminants (sheep, *Ovis aries*, accession number XP_004006224.1; goat, *Capra hircus*, accession number XP_017910309.1; cow, *Bos taurus*, accession number Q3ZBY0; camel, *Camelus ferus*, accession number XP_032325583.1) and some other common species (horse, *Equus caballus*, accession number NC_009158; pig, *Sus scrofa*, accession number XP_003124869.4; dog, *Canis familiaris*, accession number Q28296; cat, *Felix catus*, accession number XP_003984317.1; mouse, *Mus musculus*, accession number O09198; rat, *Rattus norvegicus*, accession number Q64349). (**B**) Schematics showing that, whereas the *Clostridial* botulinum (BoTN) and tetanus (TeTN) toxins target the indicated SNAREs, ETX appears to target MAL.

It is notable that CHO cells, which lack endogenous MAL, expressing exogenous rat MAL show a 10-fold increase in binding to ETX compared to those expressing human MAL [152]. Therefore, species-specific differences in the MAL sequence contribute to the remarkable differences in toxin-mediated pathologies observed between species, and make humans virtually asymptomatic for the toxin. It is of note that the integrity of the last extracellular loop of MAL is necessary for ETX binding to the cells and for cytotoxicity [152]. Therefore, this loop was proposed to form part of the site of ETX binding. However, although there are some species-specific differences in the primary sequence of the loop between human MAL and that of common mammals of domestic or economic interest (Figure 3A), the loop is identical in humans and rats. This observation suggests that, although the last extracellular loop of MAL could contribute to ETX binding, species-specific differences in other parts of MAL might be also important (Figure S1). This differential sensitivity should be considered when developing vaccines based on epsilon toxin mutants [155].

Large-scale hemolysis occurs, sometimes producing severe anemia, in patients infected with *C. perfringens* [160,161,162]. ETX causes hemolysis in humans but not in other mammals [163], suggesting that ETX-binding receptors are present on human red blood cells but absent from those of other species. Consistent with these findings, a shorter 11-kDa MAL isoform (Figure S2) was found in human erythrocytes but not in those of mouse or rat [164]. This isoform, which, given its size, might be devoid of exon III-encoded sequences, appears to be the MAL species expressed by human erythrocytes [165,166]. These results are further evidence of MAL being the cellular receptor for ETX and they identify MAL as a potential target to prevent ETX-mediated erythrocyte damage in the case of *C. perfringens* infection of humans.

ETX is the third most potent toxin after botulinum and tetanus toxins [167]. The latter are proteases that hydrolyze SNAREs (VAMP/synaptobrevin, SNAP25 and syntaxin) [168,169], which are proteins involved in membrane fusion during vesicular delivery of cargo to the corresponding membrane [170,171]. Consistent with its known role in vesicular transport, MAL joins SNARES as a novel membrane trafficking target of Clostridial toxins (Figure 3B). Despite this parallelism, it should be noted that botulinum and tetanus neurotoxins, which act on nerve terminals, have a different mechanism of action and are involved in different membrane trafficking compartments than ETX.

## 9. MAL as Organizer of Condensed Membranes for Specialized Processes

Although the high-density packing of saturated lipids and cholesterol in membranes provides the biophysical basis for membrane compartmentalization, condensed membranes require specialized protein machinery to organize these lipids into discernible subcellular structures and/or to ensure their functionality. For instance, caveolins organize condensed domains into highly organized structures named caveolae, which are small flask-shaped invaginations (60–100 nm in diameter) of the plasma membrane that are involved in signal transduction and intracellular transport. Whereas 40–60% caveolin-1 fractionates into DRMs [55], 100% MAL does this [98,172], indicating that MAL has a very strong propensity for associating with this type of membrane. MAL and caveolin-1 do not associate [5], and condensed membranes containing MAL or caveolin show distinct resistance to detergent solubilization [5,173] and are structured distinctly: caveolin-1 is found in caveolae and in flat membranes with no distinguishable morphology [174], whereas MAL is present only in the latter type of membrane [25,72]. Caveolin-1 and cavin-1, which, like caveolin-1, is a structural component of caveolae, individually sort distinct plasma membrane lipids, and together generate a unique lipid nano-environment selective for specific lipid headgroups and acyl chains. It has been proposed that this membrane remodeling allows caveolae to act as hubs that direct and guide lipid metabolism, vesicular trafficking, and signaling [175].

There is compelling evidence from various cell systems that MAL is active in the organization of condensed membranes to make them functional:

(1) In polarized epithelial cells, HA is sorted to the apical surface and partitions into DRMs in control cells, whereas HA transport to the apical membrane is blocked and the association of HA with DRMs is decreased in MAL KD cells [59].
(2) The exogenous expression of MAL in epithelial A489 cells, which lack endogenous MAL, increases the presence of HA into DRMs [59]. In addition, mutations that prevent HA from being isolated in DRMs are more weakly associated with MAL [176].
(3) The base of primary cilia is highly condensed in wild-type MDCK cells, whereas the degree of condensation is dramatically lower in MAL KD cells, resulting in fewer and shorter cilia [46].
(4) Lck is transported to the plasma membrane and partitions into DRMs in control T cells, whereas Lck is retained at the Golgi and loses its association with DRMs in the absence of MAL expression [24]. Another effect of MAL depletion in T cells is that the biogenesis of exosomes carrying GPI-anchored proteins, which normally partition into DRMs, is much less extensive than in control cells [128]. Additionally, the mistargeting of MAL from the cSMAC to the pSMAC in T cell–APC conjugates in the presence of antigen reorganizes the structure of the IS in such a way that condensed membranes and associated proteins, such as Lck and LAT, change their distribution in the same manner as MAL [45].
(5) In myelin-forming cells, neurofascin 55 distributes to the paranodal domain and is recovered from the DRM fraction in wild-type mice, but is excluded from the paranodal zone and from DRMs in MAL KO mice [119]. In oligodendrocytes, MAL overexpression interferes with the shift of PLP from a Triton X100-insoluble to a TritonX-100-soluble and CHAPS-insoluble form, impeding the switch of PLP targeting to myelin from transcytosis to lateral diffusion, which is associated with a conformational change in PLP [112,114].
(6) The defects in protein transport, membrane condensation, and protein partitioning into DRMs observed in polarized epithelial cells and T lymphocytes in MAL silenced cells are corrected by the exogenous expression of MAL [24,59].
(7) The expression of MAL in hepatic WIF-B cells, which lack endogenous MAL expression as do normal hepatocytes, increases the partitioning of specific apical proteins into DRMs and reroutes them from the indirect route to a direct pathway to the apical surface [64].
(8) ETX, which associates with condensed plasma membrane regions, binds only to cells that express MAL, and the binding is lost upon MAL KD [152,153,154]. Reciprocally, the expression of MAL in cells that do not express the protein endogenously renders condensed membranes competent for ETX binding [152,153].
(9) MAL overexpression in COS-7 cells generates giant condensed domains at the plasma membrane that contain exogenous MAL, and restricts membrane pore expansion and percolation produced by extraction with 1% Triton X-100 [44,177].
(10) Giant plasma membrane vesicles separate into coexisting ordered and disordered lipid phases [32,178]. From a set of 24 structurally and functionally diverse multipass membrane proteins, only MAL and PLP, which is a major myelin component, showed affinity for the ordered lipid phase [41]. It is of note that MAL overexpression out-competes PLP from these domains but PLP overexpression does not affect MAL partitioning. This observation was interpreted as meaning that MAL and PLP compete for a component of the ordered lipid phase present in limited amounts, and that MAL has a higher affinity for it than does PLP [41].

**Figure 4:**
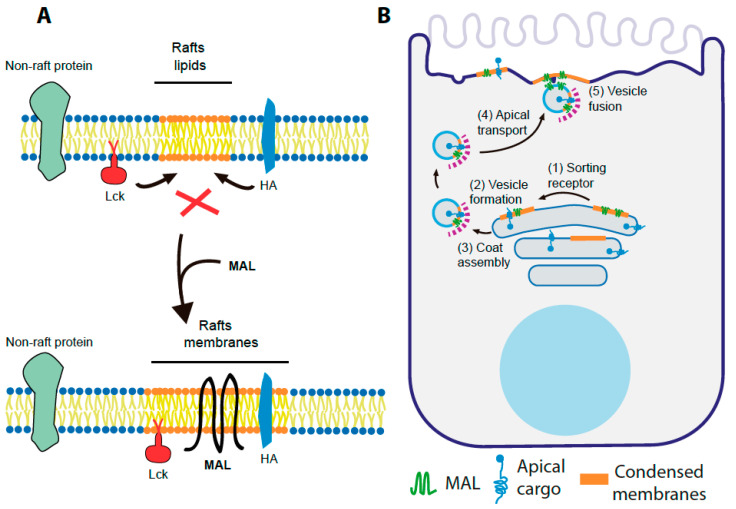
MAL as organizer of raft lipids. (**A**) MAL remodels raft lipids to generate specialized membranes functionally competent for the recruitment of cargo molecules. (**B**) Model of MAL function in apical transport: (1) sorting receptor; (2) vesicle formation; ( 3) coat assembly; (4) apical transport; (5) vesicle fusion.

Similar to the case of caveolin-1 and cavin-2 [175], the wide range of previously evaluated observations suggests that the main function of MAL is to organize membrane lipids to create specific condensed membrane environments that selectively recruit specific molecules to specialized pathways of membrane trafficking and signaling (Figure 4A). 

## 10. A Speculative Model of MAL Function in Membrane Trafficking

The overexpression of MAL in insect cells, which lack endogenous MAL, using recombinant baculoviruses, causes a very large number of vesicles to accumulate in their cytoplasm [179]. These vesicles were different in size from the caveolae-like vesicles induced by caveolin-1 expression using the same experimental system [180], and segregated from them, as seen in cells expressing the two proteins simultaneously [179]. Similar to the capacity of caveolin-1 to induce caveolae-like particles in this cell system, the finding that MAL induces extensive vesiculation could be interpreted to mean that a function of MAL is to form vesicular carriers. 

The carboxyl terminal RWKSS sequence of MAL fits with the “double lysine” consensus signal (R/K(X)KXX, where X is any amino acid), which was originally found to be involved in the retrograde transport of proteins from the Golgi apparatus to the ER in a process involving COPI coats [181,182] and later on postulated to be a signal for endocytosis [183]. In the MAL molecule, but not when appended to the carboxyl terminus of the single-span transmembrane protein CD4, the RWKSS sequence does not work as a signal for retrograde transport to the ER. However, the substitution of the two Ser residues by Ala diverts MAL to the endoplasmic reticulum [184]. These observations indicate that the RWKSS sequence can act as a canonical “double lysine” signal in protein contexts than that of MAL, and that is probably related to this type of signal. 

The ability of mutations that inactivate double lysine signals to render MAL molecules unable to direct HA to the apical surface suggests an important role for the RWKSS sequence in MAL function [25]. Although the specific steps involving the RWKSS sequence are not yet known, it could mediate MAL shuttling between different subcellular compartments and possibly facilitates the assembly of as yet unidentified vesicular coats. We propose that MAL compartmentalizes membranes at the Golgi, endosomes and plasma membrane to allow the selective recruitment of specific cargo. Through its RWKSS sequence, and probably other sorting signals, it assembles vesicular coats that lead to the formation of transport vesicles that move between different subcellular compartments to recruit and deliver specific cargo (Figure 4B). A role for MAL in vesicle fusion with the acceptor membrane is also proposed, giving the similarity of its MARVEL domain to that of synaptophysin [16] and based on the deficient fusion of plaque-transporting vesicles with the apical membrane observed in MAL KO mouse urothelium [70].

## 11. Structure of the *MAL* Gene and Promoter Region

### 11.1. The MAL Gene

**Figure 5:**
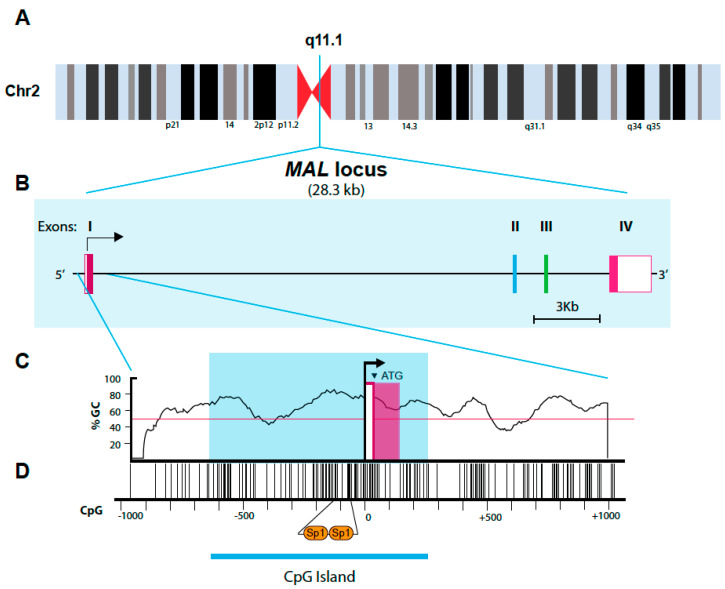
Structure of the *MAL* locus and analysis of the CpG dinucleotide content at a region surrounding the transcription start site. (**A**) The human *MAL* locus maps to chromosome 2q11.1. (**B**) Exon/intron organization of the *MAL* gene. White and colored rectangles correspond to untranslated and coding regions, respectively. (**C**) G plus C content in the indicated genomic region surrounding the transcription start site. (**D**) Distribution of CpG dinucleotides (vertical lines) in this region. The position of the transcription start site (arrow) more distal to the ATG translation initiation codon (arrowhead) is indicated. The two Sp1-binding elements are also indicated.

The human *MAL* gene spans approximately 28.3 kbp of the chromosome 2 band q11.1 (Figure 5A), and consists of four exons (Figure 5B) [185,186]. The first exon contains the entire 5’ untranslated region and the region encoding the first 31 amino acids. The second and third exons encode 56 and 42 amino acids, respectively, and the fourth exon encodes the 24 C-terminal amino acids and contains the entire 3’ untranslated region. Splicing between exons occurs in all the cases between the last nucleotide of a codon and the first of the next codon. In addition to the major transcript encoding the 153-amino acid MAL protein (sometimes referred to as MAL-a), RT-PCR analyses of human thymocytes and T cell lines indicate the existence of minor transcripts that arise from the alternative splicing of exons II and/or III (Figure S2), and that encode shorter isoforms (MAL b-d). Isoforms b and c lack the second and the third transmembrane segments, respectively, and may adopt a structure with cis-domains that loop back and exit the membrane from the same side [186].

### 11.2. The MAL Promoter

CpG islands are regions of the genome that contain a large number of CpG dinucleotide repeats (CpG observed/expected ratio > 0.6) and a G + C base content greater than 50%. In mammalian genomes, CpG islands typically extend for 500–1500 base pairs and are located within, or near, gene promoter regions [187]. Epigenetics includes any process that alters gene activity without changing the DNA sequence, and leads to modifications that can be transmitted to daughter cells [188,189]. One of the best known epigenetic process is DNA methylation, which involves the addition of a methyl moiety to the fifth carbon of a cytosine residue that typically occurs in the CpG dinucleotides [190]. In normal cells, the absence of DNA methylation within CpG-rich promoter regions is associated with active gene transcription. In cancer cells, CpG islands preceding tumor suppressor gene promoters are often hypermethylated, which results in transcriptional blockade [191,192,193]. However, the paradigm of promoter DNA methylation as a transcriptional silencing mechanism does not always hold true [194].

The 5′-flanking region of the *MAL* gene features an absence of a canonical TATA box, and the presence of a minimal promoter consisting of two binding elements for the ubiquitous nuclear factor Sp1. These elements are located close to the transcriptional start site, which is contained in an initiator-like sequence. Since the *MAL* promoter appears to lack *cis* elements responsible for its characteristic tissue-restricted pattern of expression, it was postulated that mechanisms other than tissue-specific transcription factors or specific enhancers would govern *MAL* expression [195]. It is also of note that the region within about 600 bp of the 5’ proximal promoter, the first exon, and the 200-bp sequence downstream of the first intron has a high G + C content (50.5%) and high density of CpG dinucleotides, constituting a ~0.8-kb CpG island (Figure 5C,D). 

## 12. MAL in Cancer

### 12.1. MAL Gene Hypermethylation and Silencing Are Common Features in Many Carcinomas

The analyses of the methylation status of *MAL* in normal cells and in a number of tumor specimens and tumor-derived cell lines indicate that the *MAL* CpG island is the subject of methylation, and that this epigenetic mechanism contributes to the downregulation of *MAL* gene expression in many cancers [196,197,198,199,200,201,202,203,204,205,206,207,208,209,210,211,212,213,214,215]. The use of different methods—the sequencing of bisulfite-treated DNA, methylation-specific PCR (MSP), quantitative MSP, pyrosequencing, etc. [216,217,218]—and the analysis of different segments of the *MAL* CpG island make it difficult to identify the methylation of specific CpG dinucleotides critical to the regulation of *MAL* gene expression. However, a common feature of most of these studies is that the methylation of the first exon and the promoter region most proximal to the transcriptional start site is important for regulating *MAL* gene activity. Methylation blocks *MAL* gene transcription and, consequently, greatly depletes MAL mRNA levels and silences MAL protein expression. 

In the various studies carried out, MAL mRNA levels were assayed by the hybridization of cDNA microarrays, RT-PCR, or quantitative RT-PCR. The role of methylation in *MAL* gene silencing was supported by restoring MAL mRNA levels in tumor cell lines after treatment with 5-aza-2′-deoxycytidine, which inhibits DNA methylation, combined or not with trichostatin A, which is an inhibitor of deacetylation [197,200,201,202,204,208,209,211,219,220,221]. In some studies, the ability of exogenously expressed MAL to inhibit processes related to cancer progression, such as enhanced migration, invasion or tumorigenicity, was also analyzed to confirm the involvement of MAL in the malignant phenotype [200,201,202,210,215,219]. A few studies have compared nude mice inoculated subcutaneously with tumor cells that did or did not express exogenous MAL, and found the tumor to be of smaller volume in the former group [202,210,219]. Immunohistochemical analysis of the expression and distribution of MAL in some studies confirmed the silencing of MAL expression by the hypermethylation of the *MAL* gene or was used, in the case of MAL-positive tumors, to identify neoplastic cells. Unfortunately, compared with other markers, there are few immunohistochemical assessments of MAL expression in large numbers of cancer specimens [20,200,222,223,224,225,226,227]. MAL protein detection enables more solid prognostic/diagnostic conclusions to be drawn than can be achieved by gene methylation analysis alone, which involves techniques not routinely used in most Medical Pathology Units. Table 2 summarizes the main findings from the types of tumor analyzed and shows the potential use of *MAL* gene hypermethylation/MAL protein expression as a cancer biomarker. 

### 12.2. MAL as a Cancer Biomarker 

The use of cancer biomarkers can provide information about a patient’s outcome with a rough estimate of the decrease in survival time, the type of tumor, malignant potential, risk of recurrence or response to a given therapy. Combined with the staging of the tumor as the most important criterion, the use of biomarkers can guide the selection of optimal treatment for the clinical management of the patient. There are a number of reports that establish a correlation between *MAL* gene hypermethylation/MAL protein expression and pathological characteristics of the tumors or the clinical outcome of the patients (Table 2). Thus, whereas immunohistochemical analysis showed no significant correlation of MAL protein expression in breast tumors with clinical-pathological variables or the disease-free survival period when patients received chemotherapy, a separate analysis of patients who did not receive chemotherapy showed that survival in cases of the loss of MAL expression may have been favored by adjuvant therapy [200]. In another study, *MAL* methylation frequency and methylation levels were found to be significantly higher in Barrett’s esophagus, dysplastic Barrett’s esophagus, and esophageal adenocarcinoma than in esophageal squamous cell carcinoma and normal esophagus. A significant correlation was found between *MAL* hypermethylation and Barrett’s esophagus segment length. *MAL* hypermethylation is, therefore, a common event in human esophageal adenocarcinoma but uncommon in squamous cell carcinoma [203]. In gastric cancer, hypermethylation of the proximal promoter region, but not of the distal region, is associated with better disease-free survival compared with that of patients with gastric tumors unmethylated or methylated only in the distal promoter region [199]. Epstein–Barr virus-associated gastric cancer is associated with genome-wide DNA hypermethylation of the host CpG islands, including the *MAL* gene [228]. It is interesting that the expression of exogenous MAL in gastric cancer cell lines reduces the viral titer, probably interfering with late steps of the viral replicative cycle such as the formation of viral particles or their budding from the cell [228]. These results suggest that Epstein–Barr virus-induced epigenetic silencing of the *MAL* gene benefits viral lytic replication, and facilitates viral propagation and spreading, and oncogenesis. 

In the prostate, *MAL* was identified as one of six genes whose methylation is a strong predictor of aggressive cancer in patients categorized in the group of low-to-intermediate risk disease [206]. Therefore, the analysis of the methylation of the *MAL* gene in biopsies could enhance the identification of patients whose clinical data would otherwise lead them to be considered of low or intermediate risk based on clinical variables [206,229]. MAL mRNA downregulation has been found in adenoid cystic carcinomas of salivary glands [230] and in oral squamous cell carcinoma [231]. *MAL* expression is gradually lost during progression from pre-invasive lesions to squamous cell carcinoma in tobacco-associated oral cancer [232].

The potential of MAL protein expression to discriminate between short- and long-term survival was validated by staining a larger set of advanced serous ovarian cancers unselected for disease outcome. This study showed that a high level of expression of the MAL protein is associated with shorter survival [227]. *MAL* promoter methylation and reduced MAL protein expression are suggested to be of diagnostic value for incipient colorectal tumors [208,209,213,233]. In addition, *MAL* gene hypermethylation appears to act as an independent prognostic predictor of survival advantage in postoperative patients with colorectal cancer [234]. The detection of *MAL* promoter methylation in cervical scrapings predicts the severity of cervical disease [201,205,214,215,235,236,237,238,239,240,241,242,243,244,245,246]. *MAL* is underexpressed in the group of bladder cancer patients with bad prognosis [247]. Lower levels of expression of MAL in Merkel cell carcinoma, which is a malignant neuroendocrine skin tumor that, in most cases, is associated with Merkel cell polyomavirus, are significantly associated with both unfavorable survival and the absence of Merkel cell polyomavirus in this type of carcinoma [248]. 

Cisplatin is one of the most effective chemotherapeutic agents and is used for several tumor types [249]. Epigenetic gene silencing is increasingly being recognized as contributing to the development of cisplatin resistance. The treatment with demethylating agents, such as 5-aza-2′-deoxycytidine, resensitizes patients to platinum therapy, which is evidence of the critical importance of DNA methylation in drug resistance [250,251]. MAL transcript levels are higher in cis-platinum-resistant ovarian cell lines. *MAL* methylation status and probably MAL protein expression may therefore serve as markers of platinum sensitivity [221,252].

Unlike other types of cancers in which the involvement of MAL in cancer was investigated only with respect to the methylation status of the *MAL* gene or the levels of MAL mRNA, the information about the expression of MAL in lymphomas mostly comes from immunohistochemical or flow-cytometry analysis with anti-human MAL mAb 6D9 [20,222,223,224,225,226]. MAL expression in B-cell lymphomas has been investigated in both Hodgkin and non-Hodgkin lymphomas. Most studies of the latter have been performed on primary mediastinal lymphoma, which is classified as a subtype of diffuse large B-cell lymphoma with a putative thymic origin [253,254,255]. Using differential gene expression analysis, MAL mRNA was found to be expressed in mediastinal but not peripheral diffuse lymphomas. Immunohistochemical analysis confirmed the selectivity of MAL expression, revealing that approximately 64% of primary mediastinal tumors express MAL [20,222]. There is a growing body of evidence that primary mediastinal lymphoma and classic Hodgkin lymphoma are related entities because they share clinical, histopathological, and molecular genetic features [256]. It is of note that, as revealed by immunostaining, about 19% of classic Hodgkin lymphomas expressed MAL, and its expression correlated with nodular sclerosis subtype. MAL expression in this type of lymphoma is an independent risk factor for adverse outcome [226]. Therefore, MAL expression provides additional evidence of a link between classic Hodgkin lymphoma and primary mediastinal lymphoma [225,226]. MAL expression is found in primary mediastinal large B-cell lymphomas, in plasmacytoma/myeloma, and in occasional plasma cells in normal lymphoid organs, so it was speculated that primary mediastinal large B-cell lymphomas arise from a subset of thymic B cells that express MAL at a terminal stage of differentiation [20]. 

Of the T-cell neoplasms, MAL was highly expressed in lymphoblastic tumors, whereas mature T-cell lymphomas were essentially MAL-negative [20]. In a study of patients with mycosis fungoides, which is the most common type of cutaneous T-cell lymphoma, it was found that MAL was overexpressed in interferon-α-resistant cell lines compared with interferon-α-sensitive cells. Consequently, MAL expression was associated with a longer time to remission in interferon-treated patients [223].

In conclusion, the analysis of *MAL* gene methylation, the measurement of MAL mRNA levels and the immunohistochemical detection of the MAL protein are useful diagnostic tools for defining cancer cell subpopulations that are susceptible to becoming malignant and to metastasizing, for distinguishing between different types of cancer, or for use as biomarkers with prognostic value at least in some specific types of cancer. It is also interesting that in colorectal and ovarian cancer, the analysis has also been successful when carried out on the plasma of patients obtained from small samples of blood [257]. 

### 12.3. MAL as a Tumor Suppressor Protein

Tumor suppressor genes encode proteins, such as retinoblastoma, p53, p16^INK4a^, BRCA1/BRCA2, APC, PTEN, etc., that prevent cancer by impeding the dysregulation of pathways necessary for controlled growth and homeostasis [258]. The hypermethylation of CpG islands of tumor suppressor genes leading to their transcriptional silencing is an early event in carcinogenesis [259]. Tumor suppressor silencing leads to cancer by affecting a large variety of cellular functions, such as proliferation, migration, apoptosis, DNA repair, etc. [260].

In a number of cell lines without endogenous MAL expression derived from different types of cancer (Table 2), the exogenous expression of MAL limits a number of cancer progression-related cell activities, such as migration in wound-healing assays, invasion in Matrigel^®^, proliferation, colony formation in soft agarose, tumorigenicity in inoculated nude mice, and increases in the level of apoptosis relative to that in the parental cells [200,201,202,210,219]. These results suggest that MAL might act as a tumor suppressor protein preventing the development of cancer. In addition, the observation that the *MAL* gene is frequently hypermethylated in most cancers, as are many very well known tumor suppressor genes, is consistent with the silencing of the *MAL* gene by hypermethylation being a necessary event in the development of cancer and with the proposed role of MAL as a tumor suppressor protein in many organs.

Flotillin, a protein associated with condensed membranes that is often used as a DRM marker, fractionates into DRMs in HCC1937 cells but not in MCF10A cells, which are two breast epithelial cell lines with and without MAL protein expression, respectively. The ectopic expression of MAL in MCF10A cells corrects the failure of flotillin to fractionate into DRMs and, importantly, significantly decreases cell motility compared to that of the parental cells [200]. Keeping in mind our model of MAL function as machinery for organizing condensed membrane environments, we propose that the MAL is a tumor suppressor in many types of cancer. This function is essential for the normal functioning of important trafficking and signaling pathways that incipient cancer cells need to subvert in order to de-differentiate, migrate and invade other tissues, thereby facilitating cancer progression.

Thymic precursor of T cells and probably some thymic B cells express MAL endogenously [19,20,222,226], as do T-cell lymphoblastic leukemic cells and primary mediastinal and some Hodgkin B-cell lymphomas [20,225,226]. In ovarian cancer, unlike most carcinomas in which the *MAL* gene is hypermethylated and there is no MAL expression, MAL is expressed and the *MAL* gene is hypomethylated at the transcription start site region, although it is methylated at an upstream region [221]. It is clear is that the proposed role of MAL as a tumor suppressor does not extend to all types of cancer, and malignancies of hematological cell types in which MAL is endogenously expressed, and at least those of the ovarian epithelium, are specifically excluded. MAL may have a tumor suppressive role in some cancers and an oncogenic role in others [293]. However, there is no direct evidence of such an oncogenic role, since the link between high levels of MAL correlate and the poor prognosis of B-cell lymphomas and ovarian cancer [226,227] does not necessarily mean that MAL induces the transformation of normal cells in neoplastic cells. In any case, while the lack of MAL might result in the loss of compaction of important platforms involved in signaling and trafficking, thereby causing their dysregulation, the excess of MAL could further compact those platforms in such a way that facilitates cancer progression.

## 13. Conclusions and Future Work

It has been more than 30 years since the first report on the *MAL* gene [1]. Since then, very considerable advances have been made to understand the function of MAL in human T lymphocytes, polarized epithelia and myelin-forming cells. MAL has come to be recognized as an important component of the protein machinery for specialized membrane trafficking and signaling pathways. However, there is still a long way to go before its function is completely understood. 

Proteolipids, such as the Vo sector of the eukaryotic H^+^-pump V-ATPase, are thought to play a role in membrane fusion [294]. It will be interesting to investigate whether MAL is involved in the process by which the transport vesicle is fused with the recipient membrane, as it is believed to occur with synaptophysin. The proteolipid nature of MAL could be very suitable for this purpose, since fusion events involve membrane regions enriched in lipids characteristic of condensed domains [295]. In the case of urothelial cells, MAL KO mice apparently compensate the problem in the targeting of urothelial plaque components to the apical surface caused by the lack of MAL expression by increasing the number of transport vesicles that fuse, albeit inefficiently, with the plasma membrane [70]. It will worthwhile studying how other epithelia whose apical transport relies on MAL have circumvented its absence. Little is known about the structure of the vesicles that transport proteins with affinity for DRMs to the plasma membrane. In this regard, it will be interesting to investigate the exact role of the MAL C-terminal RWKSS sequence in the traffic of transport vesicles, to establish whether it is capable of assembling a coat, and to determine what type of coat, if any, is involved. To better understand the involvement of MAL in exosome biogenesis, it is important to elucidate whether MAL organizes condensed membranes to form intraluminal vesicles or to sort specific cargo [130]. The involvement of MAL in the binding of ETX to the plasma membrane is well documented, but its role as an ETX receptor is less clear because no direct ETX–MAL interaction has yet been observed [152,153]. It is not clear why MAL silencing affects myelin in the CNS and the PNS in a different manner despite the fact that it is normally expressed by the two types of myelin-forming cells [119,120]. It is also puzzling that, despite the importance of MAL in membrane trafficking in human T cells, MAL is absent in mouse T cells [24]. This observation raises the possibility that a compensatory mechanism exists in these cells that has not been yet identified. It also reminds us that, although animal models are undoubtedly quite useful for biomedical research, studies in human cells are essential if we are to learn about human cell functioning. 

It is a challenge to find out how MAL organizes lipids to render membranes competent for specific protein recruitment. In vitro reconstitution experiments of purified components and the use of novel approaches are needed to answer this question. It will be highly worthwhile to determine how the absence of MAL expression affects cellular membranes in order to understand the tumor suppressor role of MAL and the mechanism by which MAL silencing modifies the cell to facilitate cancer progression. In this regard, although MAL KO mice appear normal in general and have a normal life span [119], it will be interesting to compare whether they are more prone to developing cancer than wild-type mice when treated with carcinogens.

Analyzing gene methylation or measuring mRNA levels are not routine practices in most Medical Pathology Departments, since histochemistry and immunohistochemistry are the gold standards for cancer cell characterization. *MAL* gene hypermethylation or reduced MAL mRNA or protein levels are related common events during the initiation and progression of epithelial cancers. Incorporating anti-MAL antibodies into current antibody panels to detect MAL, either in biopsies or, as a non-invasive procedure, in human body fluids or waste matter [296,297], might provide a prognostic/diagnostic tool for cancer patients that can be used in routine clinical practice [257,261,270,286,287]. 

## Figures and Tables

**Table 1:** Distribution of MAL in some human tissues.

Tissue/Organ	Cells Positive for MAL Expression
Esophagus	Stratified squamous epithelium
Stomach	Surface mucus cells, parietal cells, chief cells
Small intestine	Enterocytes, crypt cells, Paneth cells
Large intestine	Mucus cells
Pancreas	Acinar cells, ductal cells, endocrine cells
Kidney	Distal convoluted tubules, collecting tubules, loop of Henle
Bladder/ureter	Superficial cells from transitional epithelium
Prostate	Ductal and acinar cells
Thymus	Cortical thymocytes, medullary thymocytes, Hassall’s corpuscles
Lymph node	T cells, high endothelial cells venules endothelium
Bronchi/trachea	Respiratory epithelium, goblet cells
Lung	Type 2, pneumocytes, mucus cells
Thyroid	Thyrocytes
Testis	Leidy cells, Sertoli cells

**Table 2:** Regulation of *MAL* gene expression in cancer.

Cancer	*MAL* Gene Hyper Methylation	Silenced MAL mRNA Expression	MAL mRNA Expression Rescue by DAC * Treatment	Down-Regulation of MAL Protein Expression (IHC ** Analysis)	Decreased Tumor Size after Exogenous Expression of MAL	Decreased Tumor Cell Functions after Exogenous Expression of MAL	References
Breast	Yes	Yes	Yes	Yes		Yes	[200,207,261]
Esophagus	Yes	Yes	Yes	Yes	Yes	Yes	[203,219,262,263,264,265,266,267]
Stomach	Yes	Yes	Yes				[199,211,228,268]
Colon	Yes	Yes	Yes	Yes			[196,197,198,208,209,213,234,257,269,270]
Ovary	No	No		Yes			[212,221,227,250,252,271,272,273]
Cervix	Yes	Yes		Yes		Yes	[201,205,214,215,235,236,237,238,239,240,241,242,243,244,245,246,274,275,276,277]
Head and Neck	Yes	Yes	Yes	Yes	Yes	Yes	[202,220,230,231,232,278,279,280,281,282,283,284,285]
Lung	Yes				Yes	Yes	[204,210]
Bladder		Yes					[247,286,287]
Prostate	Yes	Yes					[206,229]
Kidney				Yes/No ***			[19]
Skin	No	No		No			[248]
Cutaneous T-cell lymphoma		No		No			[223]
B-cell lymphoma		No		No			[20,222,224,225,226,288,289,290,291,292]

* DAC, 5-aza-2′-deoxycytidine; ** IHC, Immunohistochemical; ***, Dependent on the type of carcinoma.

## Data Availability

No new data were created or analyzed in this study. Data sharing is not applicable to this article.

## Supplementary Materials

The following are available online at https://www.mdpi.com/article/10.3390/cells10051065/s1, Figure S1: Alignment of the amino acid sequence of MAL from different species, Figure S2: Generation of MAL isoforms by alternative splicing.
