# Supplementary material for: The MAL Protein, an Integral Component of Specialized Membranes, in Normal Cells and Cancer
Source: Cells. 2021 Apr 30;10(5):1065. doi: 10.3390/cells10051065 (PMC8145151; doi:10.3390/cells10051065)
Supplement: Supplementary file 1 [file cells-10-01065-s001.zip › cells-1196434-supplementary.pdf]

```
                                                                                 LOOP1
Homo sapiens              1    MAPAAATGGSTLPSGFSVFTTLPDLLFIFEFIFGGLVWILVASSLVPWPLVQGWVMFVSV   60
Ovis aries                     ...S..S.V.S.....A....F.........V.........S..H..I..I......A..
Capra hircus                   ...S..S.V.S.....A....F.........V.........S..H..I..I......A..
Bos taurus                     ...S..S.V.S.....A....F.........V.........S..H..I..I......A..
Camelus ferus                  .P.S..S...S.....A....F.....V...V........IS.....I............
Sus scrofa                     ...S.VS...S.....A..I.F......................H..I............
Equus caballus                 ...T.SS.........A....F......L..V........I......L..L.........
Canis lupus familiaris         ......S...S..........F..................I......I............
Felis catus                    ......S.........A....F......L..V........I......I............
Mus musculus                   ......S..............F.....VC..V........I......L..A.........
Rattus novergicus              ......S............V.F..................I......M............

                                                    LOOP2                            LOOP3
Homo sapiens              61   FCFVATTTLIILYIIGAHGGETSWVTLDAAYHCTAALFYLSASVLEALATITMQDGFTYR   120
Ovis aries                     .......V.AF..V.....NR...I........V.....FG.......V..EL....F.K
Capra hircus                   .......V.AF..V.....NR...I........V.....FG.......V..QL....F.K
Bos taurus                     .......V.AF..V.....NR...I........V.S...FG........A.QL....L.K
Camelus ferus                  ....T..L.LF..V.....SG.F.I........V.S...FG......F...Q.....R.K
Sus scrofa                     ...I...A.LF..V.....SR.F.IN.......I.S...FG.......V..R....Y..K
Equus caballus                 ....G..V.LF..L...........S...V..SIT..L....A........L..N..I.K
Canis lupus familiaris         ...M...A.LV..........N...........I.................G..E.Y..K
Felis catus                    ...M...A.L....V..................V.................G..E.Y..K
Mus musculus                   .......S.M......T.......I........V.................S.F.....K
Rattus novergicus              ...L...S.MVM....T.......I........V...................F......

Homo sapiens              121   HYHENIAAVVFSYIATLLYVVHAVFSLIRWKSS   153
Ovis aries                      Y.....S......V...................
Capra hircus                    Y.....S......V...................
Bos taurus                      Y.....S......V...................
Camelus ferus                   Q.....S......TV..................
Sus scrofa                      Q.....S......VV.....I............
Equus caballus                  Y.......S....A...................
Canis lupus familiaris          Q.....S......V...................
Felis catus                     ......S......V...................
Mus musculus                    ...........A.VV..I...............
Rattus novergicus               ...........A.V......I............

■ Transmembrane domain
```

Figure S1. **Alignment of the amino acid sequence of MAL from different species**. The entire amino acid sequence of MAL from the species indicated in Figure 3A is shown.

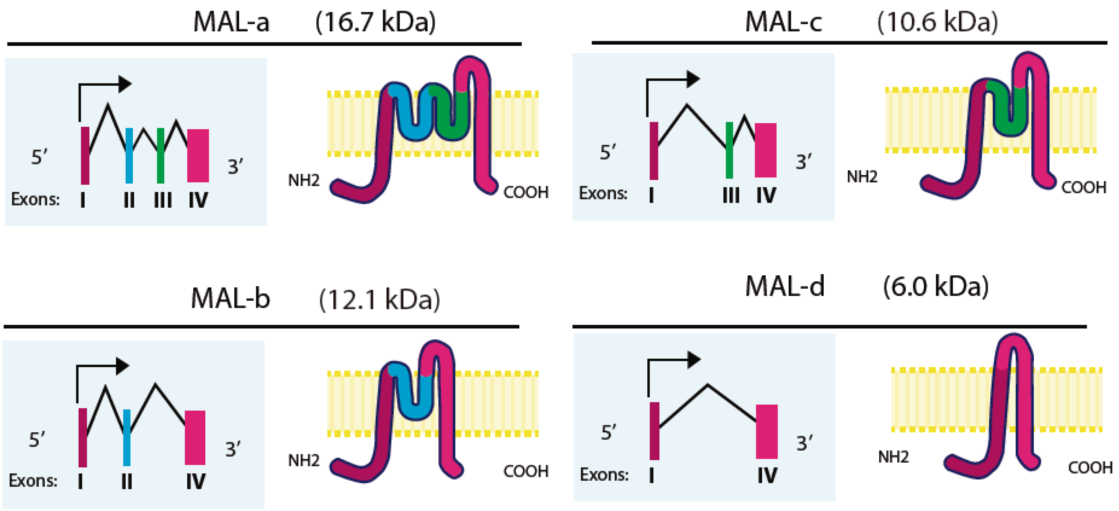

Figure S2. **Generation of MAL isoforms by alternative splicing**. Alternative splicing of exon II and /or III generates mRNA species coding for isoforms MAL b-d. Combination of the four exons encoding the MAL/MAL-a isoform. A structure of MAL/MAL-a with membrane cis-domains alternative to that containing a tetraspanning architecture is included.
